# Advanced Protective Films Based on Binary ZnO-NiO@polyaniline Nanocomposite for Acidic Chloride Steel Corrosion: An Integrated Study of Theoretical and Practical Investigations

**DOI:** 10.3390/polym14214734

**Published:** 2022-11-04

**Authors:** May Ahmed Al-Masoud, Mai M. Khalaf, Fakiha El-Taib Heakal, Mohamed Gouda, Ibrahim M. A. Mohamed, Kamal Shalabi, Hany M. Abd El-Lateef

**Affiliations:** 1 Department of Chemistry, College of Science, King Faisal University, Al-Ahsa 31982, Saudi Arabia; 2 Department of Chemistry, Faculty of Science, Sohag University, Sohag 82524, Egypt; 3 Chemistry Department, Faculty of Science, Cairo University, Giza 12613, Egypt; 4 Department of Chemistry, College of Science and Humanities in Al-Kharj, Prince Sattam bin Abdul-Aziz University, Al-Kharj 11942, Saudi Arabia; 5 Chemistry Department, Faculty of Science, Mansoura University, Mansoura 11432, Egypt

**Keywords:** polymeric composites, corrosion protection, Monte Carlo simulations, acidic chloride-induced corrosion

## Abstract

Due to their thermal stability characteristics, polymer/composite materials have typically been employed as corrosion inhibitors in a variety of industries, including the maritime, oil, and engineering sectors. Herein, protective films based on binary ZnO-NiO@polyaniline (ZnNiO@PANE) nanocomposite were intended with a respectable yield. The produced nanocomposite was described using a variety of spectroscopic characterization methods, including dynamic light scattering (DLS), ultraviolet–visible spectroscopy (UV-Vis), Fourier-transform infrared spectroscopy (FTIR), and X-ray photoelectron spectroscopy (XPS) approaches, in addition to other physicochemical methods, including X-ray powder diffraction (XRD), transmission Electron Microscopy (TEM), field emission scanning electron microscopy (FESEM), and selected area electron diffraction (SAED). By using open-circuit potentials (OCP) vs. time, electrochemical impedance spectroscopic (EIS), and potentiodynamic polarization (PDP) methods, the inhibitory effects of individual PANE and ZnNiO@PANE on the mild steel alloy corrosion in HCl/NaCl solution were assessed. The ZnNiO@PANE composite performed as mixed-type inhibitors, according to PDP findings. PANE polymer and ZnNiO@PANE composite at an optimal dose of 200 mg/L each produced protective abilities of 84.64% and 97.89%, respectively. The Langmuir isotherm model is used to explain the adsorption of ZnNiO@PANE onto MS alloy. DFT calculations showed that the prepared materials’ efficiency accurately reflects their ability to contribute electrons, whereas Monte Carlo (MC) simulations showed that the suitability and extent of adsorption of the ZnNiO@PANE molecule at the metal interface determine the materials’ corrosion protection process.

## 1. Introduction

Metal corrosion is a costly scientific issue that appeared with the beginning of metal detection [1]. It has a significant economic and environmental influence on the worldwide infrastructure causing huge losses in time and money due to the periodic maintenance work. One of the most used materials in infrastructure is carbon steel, owing to its strength, solidity, and versatility. However, the surrounding environment induces the occurrence of electrochemical reactions, which frequently damage metallic materials. Thus, it was necessary to look for practical solutions to prevent or at least reduce the environmental impact on the metallic system [2]. Several methods were explored to extend the useful lifetime of carbon steel such as cathodic and anodic protection, coatings, and corrosion inhibitors [3]. In addition, corrosion inhibitors were introduced recently on biodegradable Mg implants [4,5] and Mg-alloys [6,7] in addition to general biomedical applications [8].

Corrosion inhibitors have significant importance in the corrosion protection field, they are available, easy to use, and cost-effective [9]. At present, conducting polymers (CPs) are attracting great interest in corrosion protection since they can deactivate the metal surface with protective oxide film formation, besides their ability to use as inhibitors in the corrosive medium. Polyaniline (PANE), polypyrrole (PPy), and polythiophene (PTh) are the main examples belonging to conducting polymers [10]. Polyaniline (PANE) attracted more consideration due to its low cost, high stability, good anti-corrosion feature, and premium electrical and optical qualities, which guarantee its broad use in several technological and commercial fields [11].

At the same time, the pure polymer is not appropriate for the practical requirements of corrosion protection, in which some properties such as porosity and anion exchange of CPs could be unsuitable, particularly when it comes to corrosion made by small aggressive anions such as chlorides [12]. Therefore, some strategies have been examined to avoid the flaws in CP. One of the effective strategies is to consider CP-based composites that usually include CP where various inorganic fillers such as metal oxides were encapsulated. In other words, filler particles are dispersed completely in the polymer matrix in this type of material [13,14,15,16].

Nanoparticles (NPs) of alumina, titania, and silica are well-known materials used as fillers. They all behave as good water barriers, so they prevent the material absorption of water and prolong the service life of metals. They can be added either to the coatings or to the corrosive media in specific quantities to enhance their performance [2,17]. For instance, nickel oxide is considered a suitable solid particle for anticorrosion applications. It is known as an effective material in the industry for preventing the oxidation of metallic surfaces [18,19].

Yu-Jun et al. reported that Ni–CeO_2_ composite coatings have superior tribological performance [20]. A comparison of anticorrosive studies of spray-coated TiO_2_ and NiO thin films has been performed by Shajudheen et al. [21]. Deng et al. have stated the corrosion behavior of electrodeposited Ni and Cu nanocrystalline foils with the effect of UV light [22]. No previous reports were achieved to use nanocomposites of bimetal oxides/polymer as corrosion inhibitors for steel alloy protection. Therefore, in this report, bi-metal zinc-nickel (ZnO/NiO) mixed oxide was applied as a core compound to synthesize ZnNiO@PANE nanocomposite and examine its corrosion protection features.

ZnNiO@PANE nanocomposite was investigated as a protective film for steel corrosion in an acidic chloride medium. The prepared composite was structurally characterized using numerous spectroscopic methods including XRD, UV-Vis, FTIR, FESEM, HR-TEM, DLS, and XPS tools. The corrosion protective features of the prepared ZnNiO@PANE nanocomposite were assessed electrochemically by using *E*_OCP_, EIS, and PDP. Adsorption isotherm models were tested to explore the adsorption mechanism of ZnNiO@PANE inhibitor on MS alloy surface. Additionally, simulation analysis based on DFT calculations and MC simulations was completed to provide more information on the inhibition mechanism of the ZnNiO@PANE composite.

## 2. Experimental Part

### 2.1. Solutions, Materials, and Sample Preparation

Zinc nitrate hexahydrate (Zn (NO_3_)_2_·6H_2_O, with 99% purity sodium dodecyl sulphate (SDS) (≥99.0%), Potassium hydroxide, ACS reagent (≥85%) pellets, absolute ethyl alcohol (99%), ammonia (NH_3_) (33%), urea (NH_2_CONH_2_) (≥99%) pellets, aniline (≥99.5%), 37% HCl (Merck), potassium persulfate KPS (K_2_S_2_O_8_) (≥98.0%), nickel nitrate salt (Ni(NO_3_)_2_). The mild-steel (MS) has the following chemical composition (ASTM) in wt.%: 0.28% carbon, 0.14% chromium, 0.86% manganese, 0.034% phosphorus, 0.04% sulphur, 0.19% silicon, and the remainder was iron. The electrodes were covered by epoxy resin, keeping a 1.0 cm × 1.0 cm surface exposed to the corrosive medium. Before the electrochemical and surface analytical experiments, the MS alloy surface was polished to a mirror finish using several grades of 320, 400, 600, and 1500 grits sandpaper, washed with anhydrous ethanol, and dried in the air [18]. A standard solution of 5000.0 mg/L was made in 50.0 mL of the aggressive solution (1.0 M HCl containing 3.5% NaCl) using 1.0 g of the solid ZnNiO@PANE. For the corrosion inhibition experiments, the applied dosages of 25.0, 50.0, 75.0, 100.0, 150.0, and 200.0 mg/L were created by sufficient dilution. All chemicals were of A.R grade (Sigma-Aldrich) and used without further purifying.

### 2.2. Fabrication of ZnO-NiO Nanoparticles

#### 2.2.1. Fabrication of ZnO Nanoparticles

For the preparation of ZnO nanoparticles by the sol-gel method, a specific amount of Zn(NO_3_)_2_ was dissolved in 20 mL of bi-distilled water in a beaker. The acquired solution was stirred at 450 rpm for a few minutes and kept under stirring until dissolution is completed. On the other side, a hydrolysis process was performed by dissolving 0.3 M KOH in 100 mL of bi-distilled H_2_O. To the same Zn beaker, 1.5 M of the fuel solution (SDS + urea) was added under continuous stirring, while keeping the mass ratio of fuel: Zn near to the unity. The fuel slows down the nanoparticle’s growth rate. The pH of the obtained solution was adjusted at pH 7–8 using a known amount of (0.3 M) KOH solution, which was added slowly under stirring producing Zn sol. The solution was then left under vigorous stirring for 2 h [19]. Then, the obtained solution was sonicated at 40 °C for 40 min. Later, the solution was filtrated and washed with bi-distilled water. The obtained precipitate was dried in a vacuum oven at 110 °C for 3 h, then ground well to get ZnO nanoparticles (Figure S1).

#### 2.2.2. Fabrication of Binary Metal Oxide ZnO-NiO Nanoparticles

In a separated beaker, a known mass of Ni(NO_3_)_2_ was dissolved in bi-distilled water. Then, add the fuel solution of SDS and urea dissolved in bi-distilled water to the previous solution followed by continuous stirring until the dissolution is complete. The pH of the obtained NiO solution was adjusted at 7.5–8.5 by 33% NH_3_, added slowly under stirring. Then, the solution was left under stirring for 2 h, giving NiO sol. Finally, the NiO sol was added to ZnO sol by dropping slowly under stirring and kept under vigorous stirring for a further 1 h. The obtained ZnO-NiO blend was ultrasonicated at 40 °C for 40 min. Then, it was filtrated and washed with bi-distilled water. The obtained binary mixed oxides were dried in an oven at 130 °C for 3 h, then ground well to get ZnO-NiO nanopowders (Figure S2).

#### 2.2.3. Fabrication of ZnO-NiO/PANE Nanocomposites

ZnO-NiO/PANE nanocomposite was prepared according to the described approaches [9,23,24,25,26,27,28,29] using the route of oxidation in situ-polymerization, whereby 0.7 g of the acquired ZnO-NiO powder (prepared previously) was dispersed in 2:3 (*v:v*) of EtOH and bi-distilled H_2_O. The blend was stirred at 450 rpm for a few minutes. After that, it was ultrasonicated at 35 °C for 30 min (labelled as Sol A). In another beaker, a solution of aniline (2.2 mL) in 1.0 M of HCl acid was dispersed for 30 min. Then, 2.3 g of potassium persulfate (KPS) was added to the previous mixture with continuous stirring and labelled as (Sol B). Then, (Sol B) was added slowly to the (Sol A) under stirring till a blackish green precipitate of emeraldine salt was obtained and kept under stirring for 3 h. This mixed sol was transferred into an ice soak at a low temperature (5 °C) for 12 h. Then, it was filtrated and washed with 1 M HCl, then dried in a vacuum oven at (65 °C) for 3–4 h. The collected precipitate was treated with 1 M NH_3_ under stirring to set the pH at 8 until attaining emeraldine-base of polyaniline, which has a molecular formula (C_6_H_5_N)_n_. The solution was stirred for 12 h, filtrated, and washed several times with distilled water. Then, it was dried at 65 °C for 3–4 h in a vacuum oven to get the black precipitate and converted into powder by grinding. This sample was coded as ZnNiO/PANE (Figure S3).

### 2.3. Characterization Techniques

The surface morphology of the prepared ZnNiO/PANE composite was screened by scanning electron microscopy (SEM, Joel Jsm6360LA-Japan). After that, a transmission electron microscope (TEM) using a Jeol-1230 electron microscope was applied to describe the TEM morphology of the designed composite. Furthermore, the dynamic light scattering technique (DLS) nano Zetasizer (model: Zetasizer nano ZS90 Malvern Instruments, UK) was used to describe the scale size of the prepared particles. The X-ray diffraction (XRD, TD-3500 diffractometer) was used to analyse the crystallinity of the synthesized oxide/polymer composite at room conditions, 40 kV and 30 mA with Ni-filtered CuKα radiation (λ = 1.5418 Å). The BET surface area was studied via N_2_ adsorption-desorption isotherm and additionally used to describe the porosity of the prepared composite using Brunauer-Emmett-Teller (BET) at 77 K (Tristar II 3020 version 3.02, USA). The UV–Vis spectroscopy and FTIR spectroscopy were applied by a Shimadzu UPVC-1800 spectrophotometer (Kyoto, Japan) and BRUKER equipment, respectively. The surface chemistry of the synthesized ZnNiO-PANE composite sample was evaluated via XPS (XPS, Escalab 250Xi, Thermo Scientific, Waltham, MA, USA). Thermal gravimetric analysis equipment from New Castle, DE 19720, USA, was employed to examine the thermal stability of the prepared ZnNiO-PANE composite by increasing the environment temperature up to 1000 °C.

### 2.4. Electrochemical Analysis

Electrochemical analysis was applied using the Gamry Galvanostat/Potentiostat/ZRA electrochemical workstation. A traditional three-electrode system was utilized in this work, encompassing the Pt wire counter electrode, the MS alloy working electrode, and the silver/silver chloride (Ag/AgCl) reference electrode. Water bath heating was used to perform electrochemical tests at 298 K. The open circuit potential (*E*_OCP_) testing was done after 60 min in HCl/NaCl solution, to assure that the electrode surface had achieved a stable state. Then, the EIS test was performed at an amplitude of 10 mV and a frequency range of 100 kHz down to 0.1 Hz. Moreover, Echem Analyst software from Gamry was used to analyse the EIS data. Polarization curve testing was conducted at *E*_OCP_ ± 250 mV with a scan rate of 0.2 mV/s.

### 2.5. Computational Information

Utilizing the B3LYP-functional with DNP 4.4 basis set implemented in the Dmol3 module of the BIOVIA Materials Studio 2017, Dassault Systems, France for DFT calculations, the energy minimization of the examined PANE, ZnO@PANE, and ZnONiO@PANE compounds in aqueous conditions were evaluated [24]. The outcomes attained from DFT calculations such as the highest occupied molecular orbital (*E*_*HOMO*_), the lowest unoccupied molecular orbital (*E*_*LUMO*_), the gap energy (Δ*E*), hardness (*η*), electronegativity (*χ*), global softness (*σ*), electrophilicity index (*ω*), and Δ*E*_*back-donation*_, dipole moment (*µ*), and the number of electrons transferred (Δ*N*) were examined and calculated as follows [30]:

(1) $$\chi =\frac{-E_{HOMO}-E_{LUMO}}{2}$$

(2) $$\eta =\frac{1}{\sigma }=\frac{E_{LUMO}-E_{HOMO}}{2}$$

(3) $$\omega =\frac{\chi^{2}}{2\eta }$$

(4) $$\Delta N=\frac{\varphi -\chi_{inh}}{2(\eta_{Fe}-\eta_{inh})}$$

(5) $$\Delta E_{back-donation}=\frac{-\eta }{4}$$

where χ_*inh*_ represents the inhibitor electronegativity, *φ* is the Fe (110) function work, *η*_*Fe*_ and *η*_*inh*_ are the chemical hardness for Fe (0 eV) and additives, respectively.

Operating the adsorption locator module in the BIOVIA Materials Studio 2017, Dassault systems, France revealed the correct adsorption configurations of the PANE, ZnO@PANE, and ZnONiO@PANE compounds on the iron (110) interface for Monte Carlo (MC) simulations [31]. Firstly, the COMPASS force mild midfield was operated to optimize the adsorbate molecules [32]. Subsequently, in a simulation box (37.24 Å × 59.81 Å × 37.24 Å), the studied inhibitors, chloride ions, hydronium ions, and water molecules were successfully adsorbed onto the surface of Fe(110) [33].

## 3. Results and Discussions

### 3.1. Material Structure and Properties

#### 3.1.1. Morphology and Crystallinity Investigations

**Figure 1:**
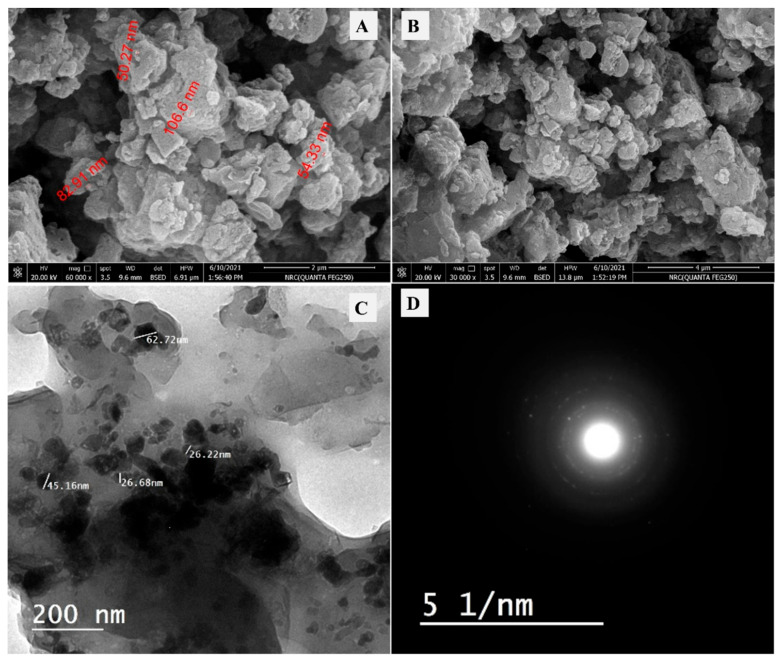
(**A**,**B**) SEM images of ZnNiO@PANE nanocomposite at different sample areas, (**C**) TEM of ZnNiO@PANE nanocomposite, (**D**) SAED of ZnNiO@PANE nanocomposite.

The morphology of the prepared ZnNiO/PANE as an oxide/polymer composite was described in Figure 1A,B. The seen morphology has an irregular size and shape of particles with some aggregated particles. The heterogeneity character of the synthesized ZnNiO/PANE composite could be due to the existence of two different parts: the polymer part in addition to the mixed oxide one having both ZnO and NiO. There are no big size pores that could be found in the SEM morphology, which could be attributed to the pores’ filling after the oxide incorporation at the surface of the applied polymer (PANE). After that, TEM and SAED analyses were studied in Figure 1C,D, respectively. Some crystalline phases could be observed which indicates the formation of crystalline oxide inside the studied composite. In addition, the presence of the polymer part that could be amorphous, causes the ambiguous character of ring circles in the shown SAED image (Figure 1D). To conclude, the morphology of the prepared ZnNiO/PANE confirmed to be a heterogeneous particle with the mix between nano and micro-scale sizes containing amorphous and crystalline parts.

**Figure 2:**
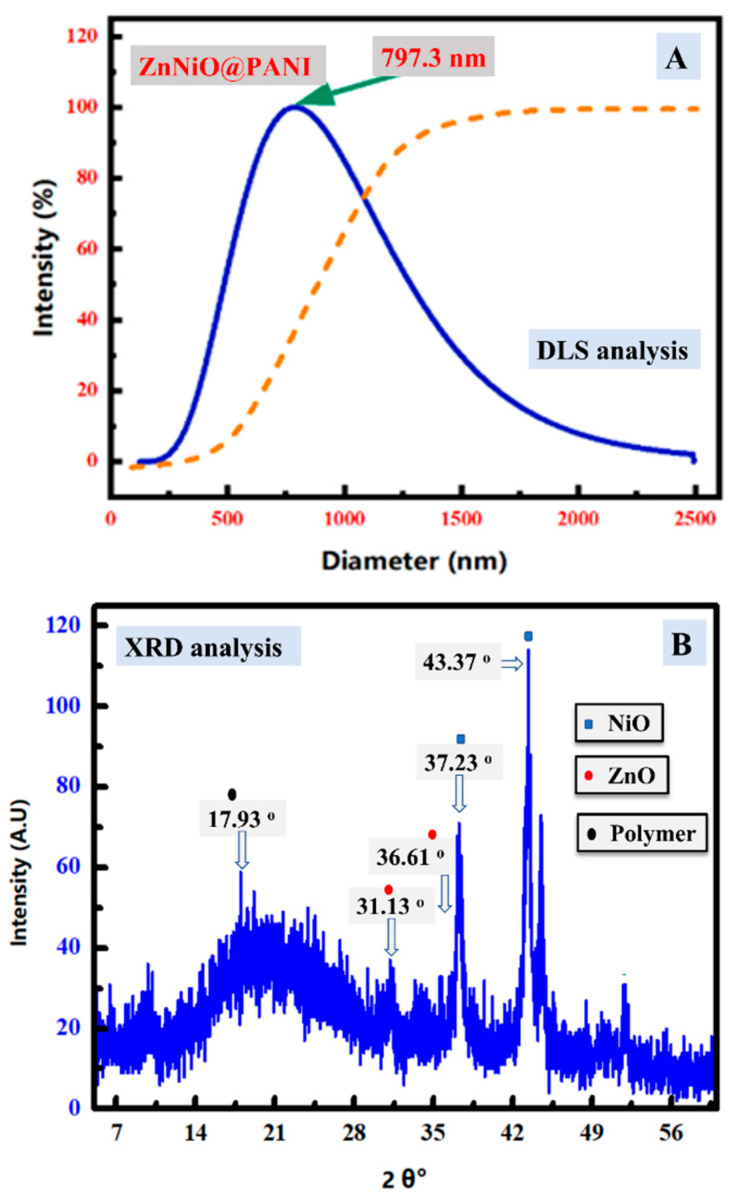
(**A**) DLS analysis of ZnNiO@PANE composite (the orange dotted line) and its differential analysis to indicate the peak (the blue line), (**B**) XRD analysis of ZnNiO@PANE composite.

The DLS curve of the designed ZnNiO@PANE composite was shown in Figure 2A. The displayed distribution size has a symmetrical and broad hydrodynamic size distribution reaching the highest size at 797.3 nm. This symmetric distribution explains the uniform dispersion of the synthesized particles, because of the stable electric dipole layer that adhered around it [34]. In addition, Figure 2B shows the XRD analysis of the synthesized ZnNiO@PANE, where only small intensity peaks could be seen that indicate the amorphous character of the prepared composite. This is likely because of the presence of polymer organic content besides the oxide part. The seen broad peak at 2*θ* = 17.93° could be assigned to the polymer cross chains. Furthermore, other small crystalline XRD peaks of NiO at 37.23°, 43.37°, and 51.95° could be due to the cubic fm-3m NiO conceded with JCPDS 02–1216 [35]. Additionally, small-intensity peaks at 31.13°, 36.61°, and 48.45° could be due to (100), (101), and (102) ZnO planes, respectively, of hexagonal crystals (space group P63mc, JCPDS 00-036-1451) [36]. This result indicates the successful incorporation of ZnO/NiO into the polymer frame to form the ZnNiO/PANE composite [37]. In short, the XRD analysis showed the amorphous character of PANE polymer as well as small content from hexagonal zincite ZnO and cubic NiO.

#### 3.1.2. XPS Analysis

**Figure 3:**
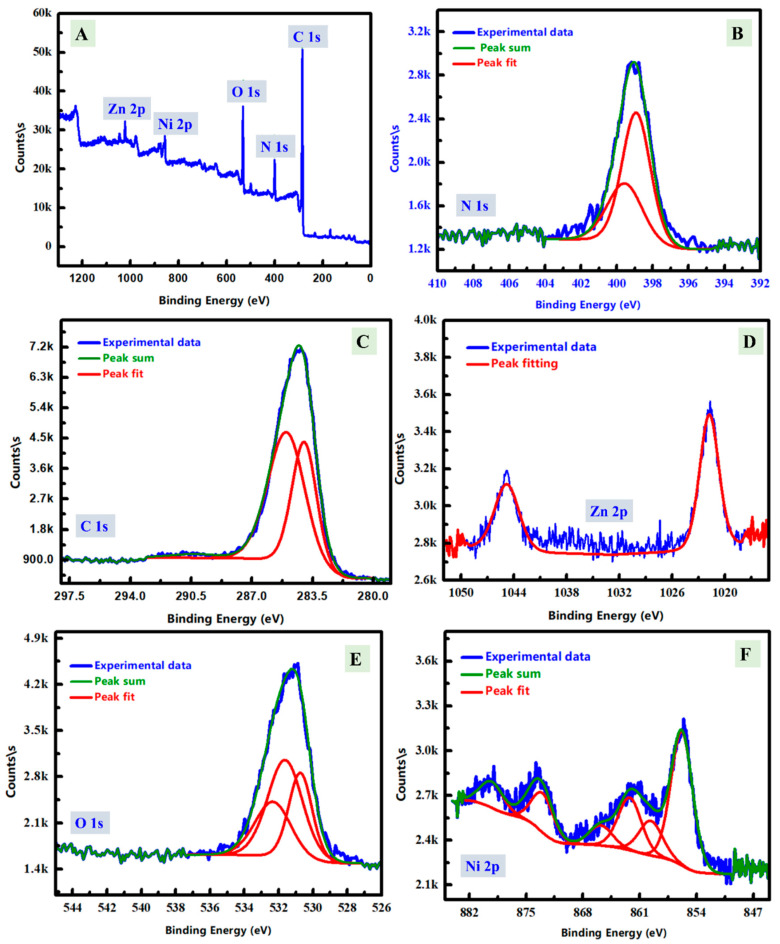
XPS scan analyses of the prepared ZnNiO@PANE composite; (**A**), and the core-level spectra of N 1 s; (**B**), C 1s (**C**), Zn 2p; (**D**), O 1s (**E**), and Ni 2p; (**F**).

To describe the chemical contents of the prepared composite surface, XPS analysis was studied as displayed in Figure 3. The survey or normal scan was shown in Figure 3A in addition to the fine scan with the fitting of N 1s, C 1s, Zn 2p, O 1s, and Ni 2p were displayed in Figure 3B–F, respectively. From Figure 3A, the XPS peaks at 283.08 eV, 402 eV, 530.08 eV, 857 eV, and 1022 eV referring to the presence of carbon (C 1s) [38], nitrogen (N 1s) [39], oxygen (O 1s) [40], nickel (Ni 2p) [38], and zinc (Zn 2p) [41]. These XPS data could suggest the existence of both ZnO/NiO oxides at the surface of the carbon content, which could be expected to be PANE polymer. The N 1s, C 1s, Zn 2p, O 1s, and Ni 2p XPS peaks were further scanned by the fine scan as shown in Figure 3B–F, respectively. For N 1s (Figure 3B), the existence of deconvoluted XPS peaks at 398.88 eV and 399.58 eV could be referred to two different nitrogen content in –C=N- and the O-M-N (M could be Zn or Ni). For C 1s (Figure 3C), the deconvoluted fitted peaks of the C1s XPS spectrum have two peaks at 283.98 eV and 285.08 eV which could be related to the carbon-carbon bond (C=C) and carbon-nitrogen bond (C-N), respectively [37,41]. Then, Figure 3D shows the fitting of the Zn 2p fine scan and two peaks could be seen at 1021.78 eV and 1044.98 eV, which can be attributed to Zn 2p_3/2_ and Zn 2p_1/2_, respectively. In addition, the binding energy separation between Zn 2p peaks was estimated and found at 23.20 eV which suggested the presence of Zn metal as Zn (+2) in the synthesized composite [41]. After that, Figure 3E described the oxygen deconvoluted peaks could be divided into three separated peaks at 530.68 eV, 531.58 eV, and 532.38 eV which confirmed the existence of different O-bonds; O-H, M-O, and C=O [42]. The deconvoluted peaks of Ni 2p (Figure 3F) at 855.78 eV and 873.48 eV for Ni 2p could be assigned to Ni 2p_3/2_ and Ni 2p_1/2_, respectively [33]. Furthermore, the satellite peaks could be seen in addition to the major peaks for Ni 2p at 879.38 eV and 859.78 eV, respectively. The binding energy separation confirmed the existence of Ni as Ni (II). The atomic % contents of N, C, Zn, O, and Ni were observed at 9.50%, 72.67%, 12.86%, 2.86% and 2.11%, respectively. Therefore, carbon is the major percentage of the prepared composite, which confirmed the polymeric character of the major content at the nanoscale surface of the designed composite. Therefore, XPS indicates the surface chemistry of the prepared composite has carbon as a major percentage in addition to O, N, Zn (II), and Ni (II) which could be due to the presence of ZnO and NiO.

#### 3.1.3. Surface Area and Thermal Gravimetric Analysis

**Figure 4:**
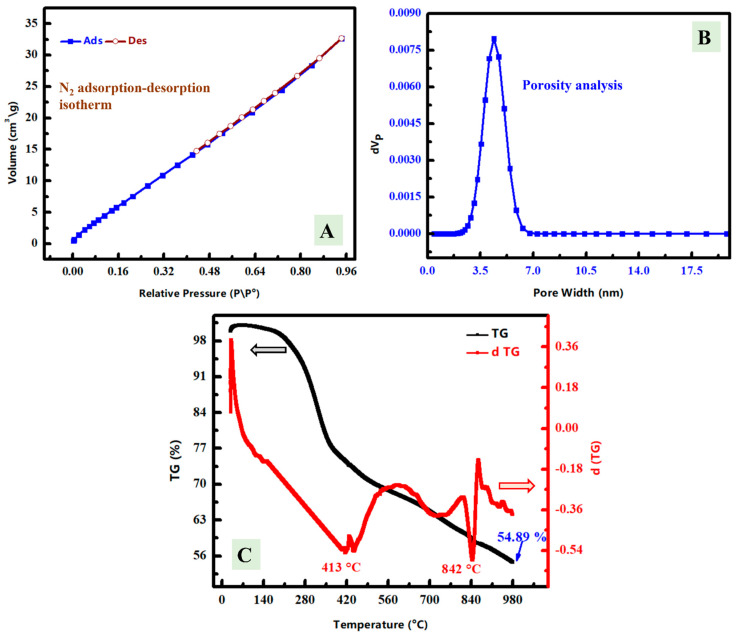
(**A**) Nitrogen adsorption-desorption analysis of the synthesized ZnNiO@PANE composite. (**B**) Pore width analysis of the synthesized ZnNiO@PANE composite. (**C**) TGA and DTG analyses of the synthesized ZnNiO@PANE composite.

To identify the porous surface capacity of ZnNiO@PANE, the surface area of the synthesized ZnNiO@PANE was analysed with an N_2_ adsorption-desorption isotherm which was displayed in Figure 4A to estimate the BET surface area at 77 K. According to the observed data, the isotherm is type IV isotherm with H3 hysteresis loop depending upon IUPAC classification, which is traditionally attributed to the predominance of mesopores. Both the adsorption and desorption curves are sloping, whereas the desorption region has a steep curve in which the remaining condensate releases suddenly from the pores as a result of the tensile strength effect [35]. S_BET_ value of ZnNiO/PANE was determined at 48.3 m^2^/g with a total pore volume of 5.05 × 10^−2^ cm^3^/g, in addition to a mean pore width at 4.18 nm (Figure 4B). The prepared ZnNiO@PANE composite has lower surface area than normal nano-oxides because of the existence of the PANE matrix with ZnO/NiO. Thus, the presence of a polymer part besides the oxide part decreases the expected surface area. Although the *S*_BET_ of the synthesized ZnNiO@PANE was low, the pores could be filled with ZnO/NiO, which can play an outstanding role as a corrosion inhibitor against the expected corrosion.

Thermal stability including TGA and DTG investigations was studied for the synthesized ZnNiO @PANE material as displayed in Figure 4C. The importance of TGA study is to determine the composition of materials, drying and ignition temperatures of the prepared materials, and knowing the stability temperatures of the introduced composite. Additionally, the introduced composite has two parts; oxide and polymer, which have different thermal stability. The weight loss at the last studied temperature (980 °C) was found at 54.89% owing to the decomposition of the organic contents (PANE polymer), which indicates the existence of more than half-percent from the prepared material as a carbon content. There are major steps of the DTG curve that can be seen. The first one is around 140 °C, which can be due to the adsorbed and crystalline water. The second and third major peaks located at 413 °C and 842 °C, respectively, could be attributed to the decomposition of organic bonds. These variable thermal gravimetric peaks for PANE decomposition might be because of its chemical structure having alkyl chains and various O-functional groups [43]. Therefore, thermal analysis indicates the existence of thermal stable content (oxide content) and decomposed one at high temperature (polymer content).

#### 3.1.4. Spectroscopic Analyses

**Figure 5:**
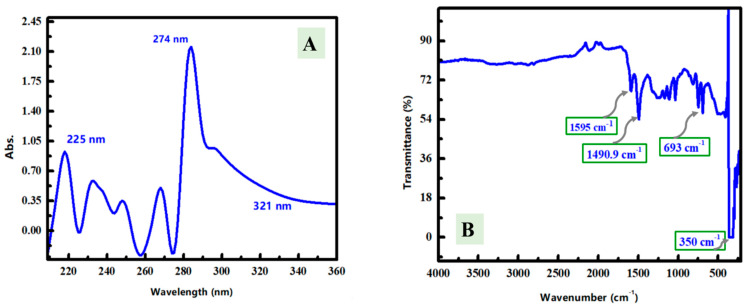
(**A**) UV–Visible spectroscopy of the synthesized ZnNiO@PANE composite in the corrosive medium. (**B**) FT-IR spectroscopy of the synthesized ZnNiO@PANE composite.

The UV–Vis spectrum was studied for the synthesized ZnNiO@PANE composite, to evaluate the optical characteristics in the corrosive medium (Figure 5A). The ZnNiO@PANE composite spectrum has an absorption peak at 225 nm that could be attributed to π-π* transition of the benzenoid ring in the organic/polymeric part. Additionally, another absorption peak was seen at around 274 nm, which could be due to the interaction between Zn/Ni oxide and PANE [9] and the absorption curve declined from 321 nm.

The functional groups and chemical bonds were studied via FT-IR spectroscopy of the prepared ZnNiO@PANE composite (Figure 5B). The FT-IR spectrum has remarkable bands of the polymer contents at 1595 cm^−1^ and 1490.9 cm^−1^ that could be assigned to the stretching vibration of C=N and bending C=C, respectively of quinonoid and benzenoid units of PANE polymer. Moreover, the FT-IR peak at 693 cm^−1^ is possibly related to the anti-symmetric stretching for M–O bonds (M may be Zn or Ni). In addition, a prominent peak can be seen around 350 cm^−1^ feasibly attributed to the out-of-plane C-H bending mode of the PANE aromatic ring [44,45]. Thus, spectroscopic analyses including UV-Vis and FT-IR analyses do confirm the interaction between Zn/Ni oxide from the first side and polymer content on the second side to finally synthesize the ZnNiO@PANE composite.

### 3.2. Corrosion Inhibition Performance

#### 3.2.1. Open Circuit Potential and Polarization Curve Analysis

**Figure 6:**
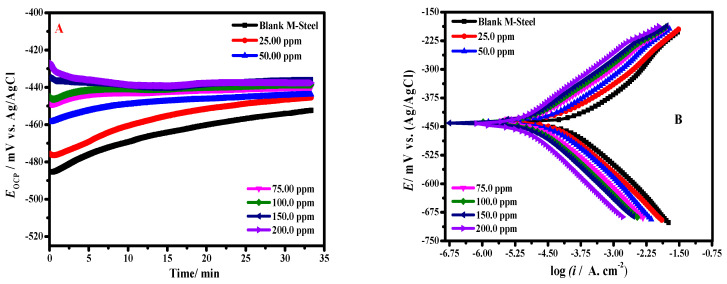
MS alloy open circuit potential vs. time (**A**) and polarization curves (**B**) in blank HCl containing brine with six concentrations (blank, 25 mg/L, 50 mg/L, 75 mg/L, 100 mg/L, 150 mg/L, and 200 mg/L) ZnNiO@PANE, and at 298 K.

The open circuit potential variation with time of MS electrode in blank HCl containing brine without and with six ZnNiO@PANE concentrations, namely, 25 mg/L, 50 mg/L, 75 mg/L, 100 mg/L, 150 mg/L, and 200 mg/L, at 298 K is depicted in Figure 6A. The data in this figure showed that the OCP inclination of the MS sample in the studied HCl saturated brine as a corrosive medium is preliminary moderately in the positive direction, demonstrating an upsurge to a slight period. In the inhibited medium containing the ZnNiO@PANE composite, the values of open circuit potential shifted towards a more positive direction throughout the soaking period compared with the blank solution. Such a behaviour can be identified by ZnNiO@PANE molecules adsorption on the MS alloy/solution interface. Subsequently, the open circuit potential values tended to be steady, indicating that the ZnNiO@PANE composite’s adsorption and desorption had attained stable operation [46]. This suggests that the system containing the ZnNiO@PANE composite had a stronger impact on the steel anodic reaction in the examined aggressive solution. The mixed metal oxide ZnO/NiO nanoparticle functionalized by PANE appeared to have high adsorption ability on the steel surface, according to the open circuit potential data.

Figure 6B illustrates the MS alloy polarization plots in HCl/NaCl solution with and deprived of ZnNiO@PANE composite. With the addition of corrosion inhibitors, it is evident that the cathodic and anodic polarization curves are modified. Among them, the current density also dropped in comparison to the blank solution. It is important to note that all the polarization curves have comparable shapes, proving that the ZnNiO@PANE composite has no impact on the corrosion process or outcomes.

The corrosion current density (*i*_*cor*_), corrosion potential (*E*_*cor*_), anodic Tafel slope (*β_a_*), and cathodic Tafel slope (*β_c_*) were all computed after the polarization data were quantitatively measured using the Tafel extrapolation method. The inhibition efficiency (*η*) and the part of surface coverage (*θ*) were also calculated as follows [47]:

(6) $$\eta (\%)=\left(\frac{i_{cor}^{0}-i_{cor}^{i}}{i_{cor}^{0}}\right)\times 100=\theta \times 100$$

where $i_{cor}^{0}$ and $i_{cor}^{i}$ stand for the MS alloy current densities in HCl/NaCl solution without and with ZnNiO@PANE composite, respectively.

The Tafel parameters for the corrosion of carbon steel in HCl/NaCl solution are shown in Table 1 for various ZnNiO@PANE composite concentrations. It is evident that *i_cor_* of the inhibited system is significantly lower than that of the blank solution. When our inhibitor is present at a dose of 200 mg/L, the *i*_*cor*_ achieves 192.66 μA/cm^2^ for individual PANE and 26.46 μA/cm^2^ for ZnNiO@PANE composite, which is appropriately lower than it would be in the blank medium (1254.34 μA/cm^2^). This indicates that ZnNiO@PANE composite can prevent electrochemical corrosion by forming an adsorbed layer on the surface of MS alloy. It is important to note that as the composite inhibitor dose is increased, the inhibition efficiency increases, suggesting that the corrosion inhibitor contributes to corrosion protection earlier on. According to Table 1, the corrosion inhibition efficiencies for individual PANE and ZnNiO@PANE are 84.64% and 97.89%, respectively, when the corrosion inhibitor concentration reaches 200 mg/L. Therefore, the optimized concentration of ZnNiO@PANE is 200 mg/L. The ZnNiO@PANE composite is adsorbed on the metal surface decreasing the number of active centres on the substrate surface, rather than changing the mechanism of anode and cathode to prevent corrosion, as can be seen in Table 1 where the modifications of cathodic (*β*_c_) and anodic (*β*_a_) Tafel slopes are not clear when compared to the blank solution data [48]. Additionally, it can be seen from Table 1 that the varied value of *E_cor_* is 16 mV, which is significantly less than 85 mV. Consequently, ZnNiO@PANE serves as a mixed inhibitor type and affects both cathodic and anodic reactions.

#### 3.2.2. EIS Measurement

**Figure 7:**
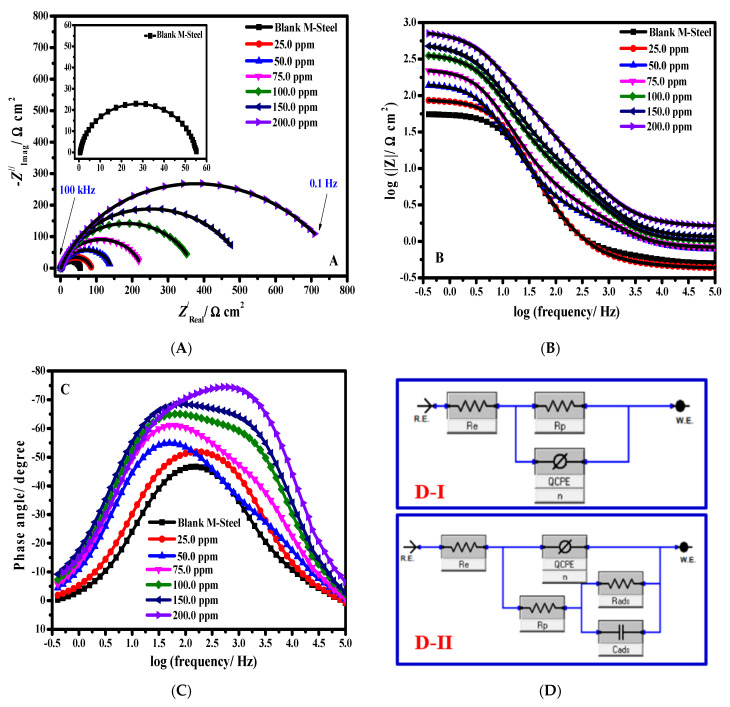
MS-alloy Nyquist plots (**A**) and Bode diagrams (**B**,**C**) in blank HCl containing brine and with six concentrations (25 mg/L, 50 mg/L, 75 mg/L, 100 mg/L, 150 mg/L, and 200 mg/L) ZnNiO@PANE, and at 298 K. Equivalent electrical circuit (**D**) in the blank (I) and inhibited systems (II).

To confirm the experimental results of the polarization curves, EIS was performed under the same conditions. The corrosion inhibition behaviour of ZnNiO@PANE in HCl-containing brine solution has been explored with EIS. Figure 7 shows MS alloy Nyquist plots (A) and Bode plots (B, C) in blank HCl containing brine without and with six ZnNiO@PANE concentrations (25 mg/L, 50 mg/L, 75 mg/L, 100 mg/L, 150 mg/L, and 200 mg/L), at 298 K. All Nyquist plots (Figure 7A) only display one capacitive loop, demonstrating that charge transfer mechanisms are solely responsible for MS-alloy corrosion. Additionally, all impedance spectra exhibited comparable forms, proving that the initial corrosion system had not been impacted by the addition of ZnNiO@PANE. The diameter of a capacitance ring expanded quickly as the concentration of ZnNiO@PANE was increased in comparison to the blank solution. Furthermore, it indicates the development of a barrier on the MS alloy surface, enhancing corrosion resistance. Typically, an MS alloy develops a passivated oxide film on its own in an alkaline environment. However, the passivation layer may deteriorate due to contamination with chlorides or carbonation, which results in pitting. The charge transfer resistance of the blank solution curve is small, as shown in Figure 7B, indicating that the passivation film is evaporating rapidly. The fact that the charge transfer resistance increased when corrosion inhibitors were present reveals that the alloy surface is protected. This occurs as a result of the active core of the organic polymeric framework adhering to the metal substrate, such as N and O, improving its ability to resist the Cl^−^ ion assault [48]. In addition, the ZnNiO@PANE protective effectiveness increases steadily with its dose in the medium. It improves MS alloy’s ability to resist corrosion.

Figure 7C displays the Bode diagrams for MS alloy immersed in HCl-containing brine without and with various ZnNiO@PANE concentrations. It is easy to see that every Bode plot has two peaks, which shows that two relaxation processes take place when the ZnNiO@PANE composite is adsorbed on the MS alloy surface. It is due to the metal’s surface adsorption of ZnNiO@PANE molecules and the resistance to charge transfer, respectively. As ZnNiO@PANE concentration was increased, the interfacial impedance value increased, indicating that the corrosion inhibitor was successful in preventing MS-alloy corrosion. It is noteworthy that the slope of the impedance modulus (|*Z*|) is −1 in the middle range of frequency, indicating the capacitive property of the adsorption of ZnNiO@PANE molecules on the steel surfaces. ZsimpWin software was used to fit the experimental data with an appropriate equivalent circuit (EC) model to quantitatively examine the inhibition efficiency of the corrosion protection [49]. In this study, Figure 7D displays the EC model in use. An element with a constant phase (*CPE*) is used in the equivalent circuit. The *CPE* is often used simply as a way to improve the fit of a model to impedance data. In this model, *R*_e_ represents the resistance of the HCl/NaCl electrolyte between the MS alloy and the reference electrodes, the polarization resistance of MS alloy is *R_P_*, *R*_*ads*_ denotes the adsorbed film resistance, and *C*_*ads*_ denotes the capacitance of the MS alloy/film interface. *Q*_*CPE*_ represents the double–layer capacitance associated with the MS alloy/solution interface. The impedance of *CPE* is defined as follows [50,51]:

(7) $$Z_{\mathrm{CPE}}=\frac{1}{Y_{0}{\left(j\omega \right)}^{n}}$$

where *Y*_0_ expresses the *CPE* admittance, *n* is a deviation index, *j* (√−1) is the imaginary number, and *ω* stands for the angular frequency (*ω* = 2π *f*). The *CPE* is equivalent to the real capacitance for *n* = 1 and the resistance for *n* = 0. Warburg impedance is not seen in this study, which also demonstrates that the ZnNiO@PANE molecules are more tightly adsorbed onto the MS alloy surface [52]. To obtain the double layer capacitance *C_dl_*, the frequency at which the imaginary component of the impedance is maximum $\left(-Z_{img}^{\prime }\right)$, is found and *C_dl_* values are calculated from the following Equation [44]:

(8) $$f(-Z_{\max}^{\prime })=\frac{1}{2\pi R_{ct}C_{dl}}$$

The impedance parameters of the MS-alloy in HCl/NaCl solution at different concentrations of ZnNiO@PANE are shown in Table 2, where *η*_E_/% is computed as follows [48]:

(9) $$\eta_{E}(\%)=\left(\frac{R_{p}^{i}-R_{p}^{0}}{R_{p}^{i}}\right)\times 100$$

The polarization resistance in an HCl/NaCl solution without and with ZnNiO@PANE is shown by the letters $R_{p}^{i}$ and $R_{p}^{0}$, respectively. The corrosion inhibition efficacy of ZnNiO@PANE increases gradually with concentration, reaching a maximum efficiency of 93.03% at the ZnNiO@PANE concentration of 200 mg/L, according to Table 2. Therefore, it has been demonstrated that ZnNiO@PANE prevents MS alloy corrosion in HCl/NaCl medium.

#### 3.2.3. Effect of Bi-Mixed Oxide ZnNiO NPs on the Inhibition Ability of the PANE

PDP tests with 200 ppm PANE and the corresponding ZnNiO@PANE nanocomposite were conducted to examine the impact of the ZnNiO NPs on the inhibitive capabilities of the PANE in the HCl/NaCl solution. The *i*_*cor*_ of the ZnNiO@PANE nanocomposite is lower than that of the consistent PANE, as shown in Table 1. This clearly demonstrates the improved protective capabilities of the examined PANE nanocomposite, which may be related to the presence of ZnNiO NPs. Table 1 shows that the presence of ZnNiO NPs caused the *i*_*cor*_ value for PANE to drop from 192.66 to 26.46 A cm^−2^. The protective capability is raised for the corresponding ZnNiO@PANE nanocomposite from 84.64% for PANE to 97.89%. This seems to suggest that the ZnNiO NPs either directly interact with the MS alloy/solution interface or promote the contribution of additional PANE active sites in the adsorption process. According to previous studies [44,45] the metal NPs have a tendency to chemisorb immediately on an MS alloy surface. At first adsorbed at the MS alloy/medium interface, ZnNiO NPs exhibit corrosion inhibition by preventing aggressive ions from reaching the metal substrate. On the layer of ZnNiO NPs, the active PANE fundamentals are adsorbed. As a result of these films’ hydrophobic character, water molecules cannot reach the MS-alloy surface.

#### 3.2.4. Adsorption Isotherm Analysis

**Figure 8:**
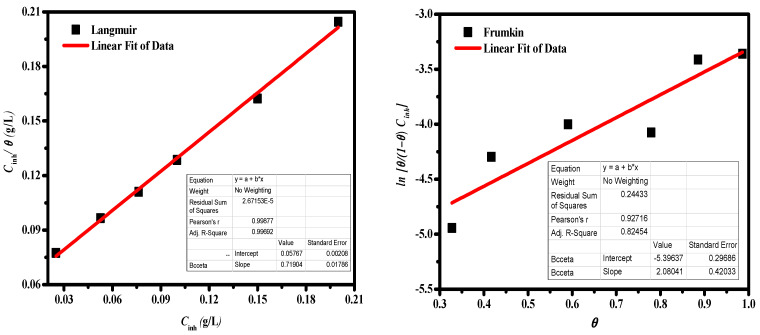

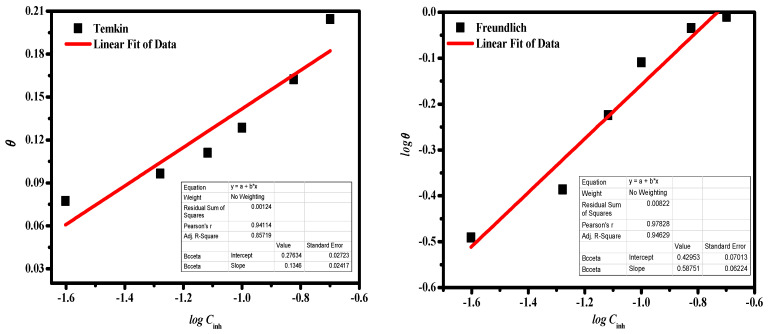
Four different adsorption isotherms for MS alloy immersed in HCl/NaCl medium and containing various concentrations of ZnNiO@PANE at 298 K.

The adsorption isotherm model was used to examine the mechanism of ZnNiO@PANE adsorption on MS-alloy surfaces. The Langmuir, Temkin, Frumkin, and Freundlich adsorption isotherm models were used to fit the experimental data of the PDP as shown in Figure 8. Where, the adsorption isotherm models are expressed in the following [53,54]:

(10) $$\mathrm{Langmuir};\Rightarrow \frac{C_{Inr.}}{\theta }=C_{Inr.}+\frac{1}{K_{ads.}}$$

(11) $$\mathrm{Temkin};\Rightarrow \exp^{\left(2\alpha \theta \right)}=K_{ads}C_{Inr.}$$

(12) $$\mathrm{Freundlich};\Rightarrow \log\theta =n\log C_{Inr.}+\log K_{ads}$$

(13) $$\mathrm{Frumkim};\Rightarrow \ln\left[\frac{\theta }{(1-\theta )C_{inh}}\right]=2\alpha \theta +\ln K_{ads}$$

where *C_inr_* describes the concentration [ZnNiO@PANE] in g/L, *K*_*ads*_ signifies the equilibrium-adsorption constant and *θ* is the surface coverage. The R^2^ values for the four adsorption isotherm models are shown in Figure 8, and the *R*^2^ value for the Langmuir isotherm model is 0.996, indicating that Langmuir adsorption offers the best fit. In general, the value of free energy of adsorption ($G_{ads}^{0}$) can be used to evaluate the type of corrosion inhibitor adsorption. The adsorption is chemisorbed when $G_{ads}^{0}$ < −40 kJ/mol. While $G_{ads}^{0}$ > −20 kJ/mol the adsorption is physisorption. The adsorption is physicochemical in case the value of $G_{ads}^{0}$ is in the range of −20 kJ/mol and −40 kJ/mol. $G_{ads}^{0}$ is calculated as follows [55]:

(14) $$\Delta G_{ads}^{0}=-RT\;\ln(1000K_{ads})$$

where 1000 is the water concentration (in g L^−1^), the *R* is the gas constant, and *T* is the absolute temperature (298.15 K) [4,23]. With the above equation, it is calculated that the $G_{ads}^{0}$ of the ZnNiO@PANE molecule is –24.21 kJ/mol. It suggests that the adsorption of ZnNiO@PANE is physicochemical [56,57]. Accordingly, the protonated ZnNiO@PANE+ molecule and the Cl^−^ ions that had previously been adsorbed on the electrode substrate (FeCl^−^ species) may interact electrostatically to cause the physisorption. Additionally, donor-acceptor interactions between the vacant d-orbitals of Fe atoms and the unshared lone pairs of electrons from nitrogen atoms and the π-electrons of aromatic rings may result in the chemical adsorption of the C-H polymer and ZnNiO@PANE composite chains on the metallic interface [58]. Long-polymer chains, therefore, have a key influence on the inhibitory and adsorption capabilities in addition to the attractions of the polymer chain with the metal interface.

### 3.3. DFT Calculations

**Figure 9:**
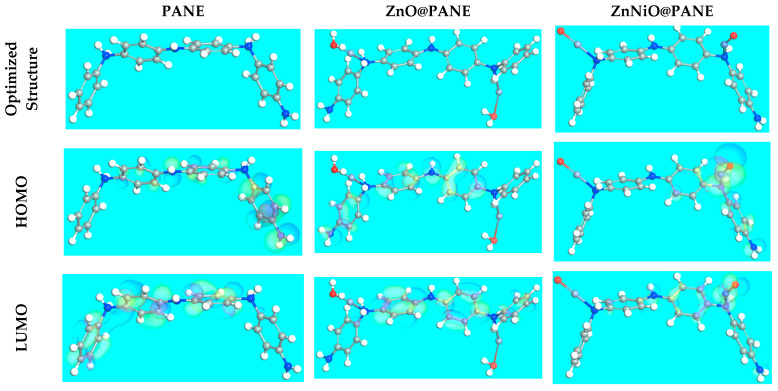
The DFT optimized structure and the occupation of the *LUMO* and *HOMO* orbitals for the PANE, ZnO@PANE, and ZnNiO@PANE compounds.

The DFT optimized structural parameters and correlated theoretical parameters for the PANE, ZnO@PANE, and ZnNiO@PANE compounds are depicted in Figure 9 and listed in Table 3. As per the FMO theory, *LUMO* and *HOMO* energies serve to identify the competence of donor or acceptor interactions performed at the surface of nanocomposite/metal [32]. Therefore, for an inhibitor molecule with low *E*_*LUMO*_ and high *E*_*HOMO*_ values, the corrosion inhibition proficiency is augmented. In comparison to PANE and ZnO@PANE compounds (−4.930, and −4.510 eV), the ZnNiO@PANE molecule has the highest *E*_*HOMO*_ value of −4.38 eV, as shown in Table 3. As presented in Figure 9, the level of *HOMO* was clearly assigned to the phenyl-amino moieties in the additive molecules, indicating that these sites are more likely to be targeted by electrophilic assaults on the CS alloy surface. These parameters support the capacity of the inhibitor additive to adsorb on the metal surface, leading to an increase in protection effectiveness that was well in line with the empirical results. On the other hand, the *E*_*LUMO*_ value is −2.51 eV for the ZnNiO@PANE composite (Table 3) lesser than PANE and ZnO@PANE compounds (−1.87, −2.03 eV). The greater protective power of the ZnNiO@PANE molecule is indicated by its lower *E*_*LUMO*_ value, which is consistent with earlier studies. Correspondingly, the energy gap (Δ*E*) is a significant stricture to improve the corrosion protection effect of the additive molecule, i.e., which upsurges as the Δ*E* value is decreased [33,59]. According to Table 3, the ZnNiO@PANE molecule has a stronger propensity to be adsorbed on the steel contact due to its slightly lower energy gap value (1.87 eV) than the PANE and ZnO@PANE molecules (3.06 and 2.46 eV).

Typically, most inhibitors have moderately low values for electronegativity (*χ*), which represents the inhibitor’s ability to contribute electrons to the surface of the CS alloy [60]. Contrarily, high values of *χ* also present a great possibility for inhibitor molecules to acquire electrons from iron surface atoms (i.e., back-donation) and form a more durable bond with the CS-alloy surface [61]. As shown in Table 3, it seems that the electronegativity for PANE, ZnO@PANE, and ZnNiO@PANE compounds is rather higher, suggesting that the compounds under consideration have the potential to donate electrons back to one another and form a more stable bond with a CS alloy interface.

Additionally, the hardness (*η*) and softness (*σ*) of an inhibitor could be utilized to gauge its reactivity and stability. Soft inhibitors, for example, have a greater shield capacity than hard compounds due to the smooth transfer of electrons to the CS alloy interface through the adsorption process, which makes them efficient corrosion inhibitors [62]. Table 3 shows that ZnNiO@PANE molecules have inferior *η* and greater *σ* values than PANE and ZnO@PANE molecules, which describes smoothly devoting electrons to the CS alloy substrate and excellent inhibitory properties. Likewise, the fraction of electron transfer and Δ*E*_*back-donation*_ are essential factors for the inhibitor’s proficiency in electron-offering or admitting. Consequently, if the Δ*N* values are more than zero, the electron relocation from the inhibitor to the steel surface atoms is likely to occur, but the electron relocation from the metal atoms to the inhibitor molecule is feasible if the Δ*N* values are less than zero (i.e., back-donation) [63]. The fact that the studied molecules had Δ*N* values greater than zero, as listed in Table 3, indicates that the PANE, ZnO@PANE, and ZnNiO@PANE molecules are sufficiently able to contribute electrons to the CS-alloy surface. Moreover, the Δ*E*_*back-donation*_ will be less than zero when *η* is more than zero, and the electron is removed to a compound, tracked by a back-donation from the molecule, and this is energetically desired [64]. Table 3 shows that the values of *E*_*back-donation*_ for PANE, ZnO@PANE, and ZnNiO@PANE are negative, indicating that back-donation is desirable for these materials and will result in a strong connection [56].

Moreover, the dipole moment is a precarious stricture that favours a predictive method of corrosion protection [65]. The increase in dipole moment improves the adsorption of the compound on the metal substrate and improves the distortion energy. Accordingly, the rise in dipole moment results in improved anticorrosion ability [66]. As revealed in Table 3, the ZnNiO@PANE compound has a larger value of dipole moment (22.41 Debye) than PANE and ZnO@PANE compounds (7.06, 12.72 Debye), which approves the more tendency for ZnNiO@PANE compound to be adsorbed on the metal surface and enhance its protection. The local reactivity of the prepared compounds could be assessed from the Fukui indices ($f_{k}^{+}$ and $f_{k}^{-}$), Mulliken atomic charges, the local electrophilicity ($\omega_{k}^{\pm }$), local softness descriptor ($\sigma_{k}^{\pm }$), and the dual descriptors ($\Delta f_{k},\Delta \sigma_{k}\;\mathrm{and}\;\Delta \omega_{k}$) from the subsequent equations [67]:

(15) $$\sigma_{k}^{\pm }=\sigma f_{k}^{\pm }$$

(16) $$\omega_{k}^{\pm }=\omega f_{k}^{\pm }$$

(17) $$\Delta f_{k}^{\pm }=f_{k}^{+}-f_{k}^{-}$$

(18) $$\Delta \sigma_{k}=\sigma_{k}^{+}-\sigma_{k}^{-}$$

(19) $$\Delta \omega_{k}=\omega_{k}^{+}-\omega_{k}^{-}$$

**Figure 10:**
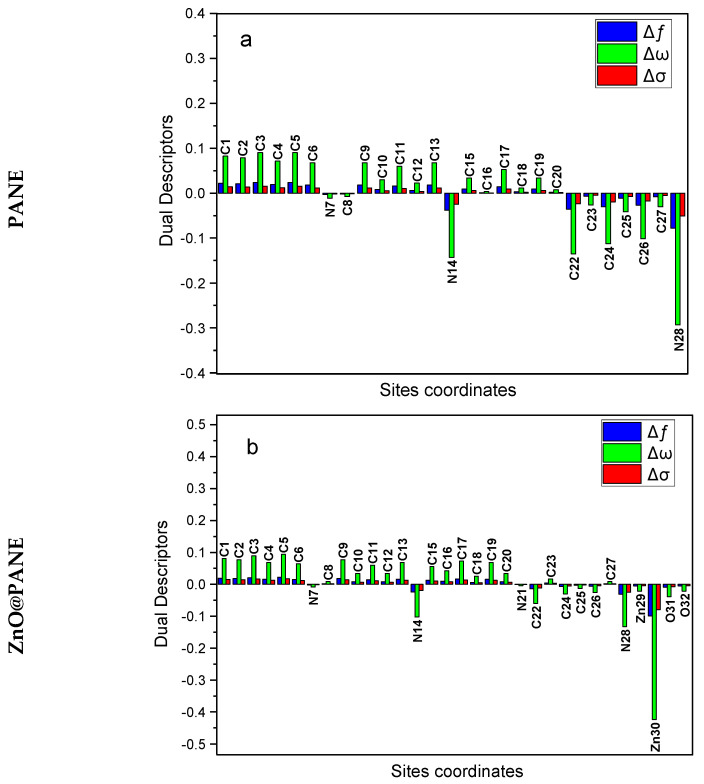

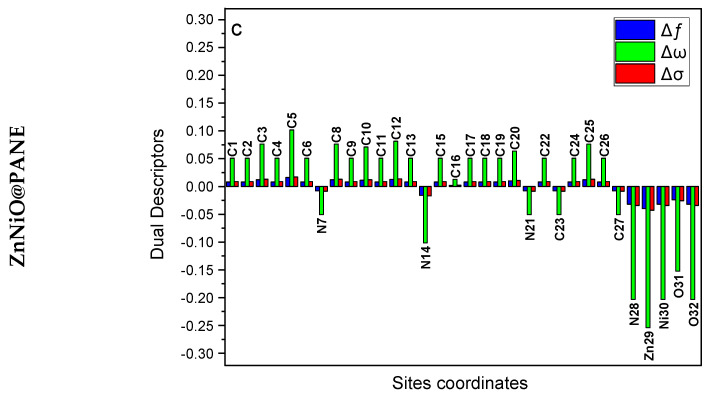
Graphical illustration of the Dual descriptors for the most efficient locations of the examined PANE, ZnO@PANE, and ZnNiO@PANE compounds.

For explanation, Table S1 presents the most important findings. The assessed Fukui indices (Table S1) identified the inhibitor molecules as well as the places where the molecules of PANE, ZnO@PANE, and ZnNiO@PANE will adsorb to the iron surface. The $f_{k}^{+}$ implies the reactivity of the nucleophilic attack centres (accepting centres) whilst, $f_{k}^{-}$ designates the electrophilic attack reactivity (donation sites) [68]. The highest $f_{k}^{-}$ for C8-13, C15-20, N14, C22-27, and N28 for PANE; C8-13, C15-20, C22-27, N14, N28, O31, and Zn30 for ZnO@PANE; and C1-6, C9-13, N14, C15-20, C22-27, N28, Ni30, Zn29, Ni30, and O31-32 for ZnNiO@PANE designate the electron contributing site. While the highest $f_{k}^{+}$ is found at C1-6, C8-13, C15-20, and C23-26 for PANE; at C1-6, C8-13, C15-20, C23-24, C26-27, N28, and Zn29-30 for ZnO@PANE; and at C1-6, C9-13, N14, C15-20, C22-27, N28, Ni30, Zn29, Ni30, and O31-32 for ZnNiO@PANE revealing the capability for a back-donation [69]. An additional measure of a molecule’s local reactivity is its Mulliken atomic charge, which is shown in Table S1 for the molecules PANE, ZnO@PANE, and ZnNiO@PANE. Higher-negative-charged atoms, which resemble electron donors (nucleophilic centres) [56]. Consequently, the atoms C1-5, N7, C9-10, C12-13, N14, C16-17, C19-20, N21, C23-24, C26-27, and N28 for PANE; C1-5, N7, C9-10, C12-13, N14, C16-17, C19-20, N21, C23-24, C26-27, Zn29, N28, O31, and O32 for ZnO@PANE; and C1-5, N7, C9-10, C12-13, N14, C15-20, N21, C23-24, C26-27, and O31-32 for ZnNiO@PANE are all active places on oxygen atoms and phenylamino moieties that can grant electrons when they interact with the surface of a metal. In addition, the local dual descriptors are further accurate tools than the Fukui indices, as well as the local softness and electrophilicity. Figure 10 displays a graphical depiction of the dual local descriptors of the most representative active locations. The attained outcomes display that the locations with the $\Delta f_{k},\Delta \sigma_{k}\;\mathrm{and}\;\Delta \omega_{k}$ < 0 have the propensity to move electrons to the steel surface. Conversely, those sites with $\Delta f_{k},\Delta \sigma_{k}\;\mathrm{and}\;\Delta \omega_{k}$ > 0 have the capability to receive an electron from the CS alloy. As can be seen in Figure 10, the active centres for electron contribution are at N7, N14, C8, C22-27, and N28 for PANE; C22, C24-27, N7, N14, N21, N28, O31, O32, and Zn29-30 for ZnNiO@PANE; and N7, N14, N21, C23, C27, N28, Zn29, Ni30, and O31-32 for ZnNiO@PANE. While the active centres for electron receiving are at C1-6, C9-13, and C15-20 for PANE; C1-6, C8-13, C15-20, C22, and C24-26 for ZnNiO@PANE; and C1-6, C19-20, and C22-27 for ZnNiO@PANE.

**Figure 11:**
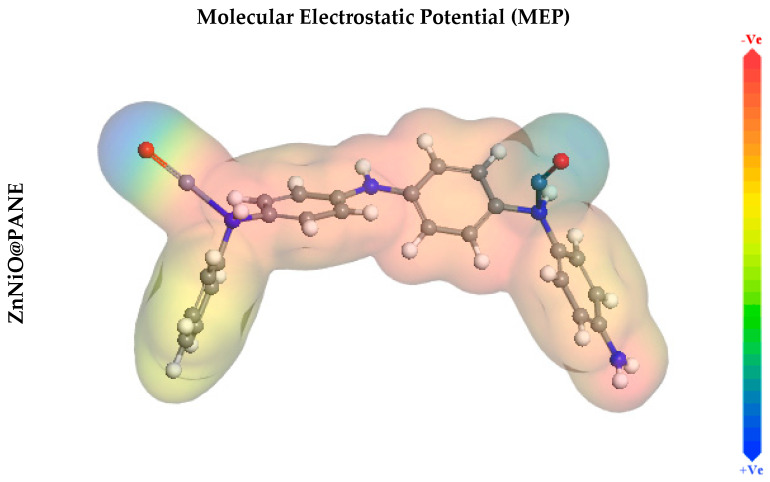
Molecular Electrostatic Potential (MEP) of the studied ZnNiO@PANE molecule.

Additionally, the Dmol^3^ module evaluates molecular electrostatic potential mapping (MEP), which may reveal the active sites of the additive molecule. The MEP mapping is a 3D visual indicator used to recognize the total charge distribution’s overall electrostatic impact on a molecule [70]. The great electron density region, where the MEP is particularly negative, is depicted by the red colours in the MEP maps shown in Figure 11 (nucleophilic reaction). Inversely, the shades of blue represent the area with the greatest positivity (electrophilic interaction) [71]. Figure 11′s optical analysis confirms that the highest negative regions are primarily above oxygen and nitrogen atoms, while the benzene rings have a lower electron density. These areas of increased electron density (i.e., the red area) in inhibitor molecules may be the utmost suitable for CS alloy surface attractions that produce strong adsorbed protective layers.

### 3.4. MC Simulations

**Figure 12:**
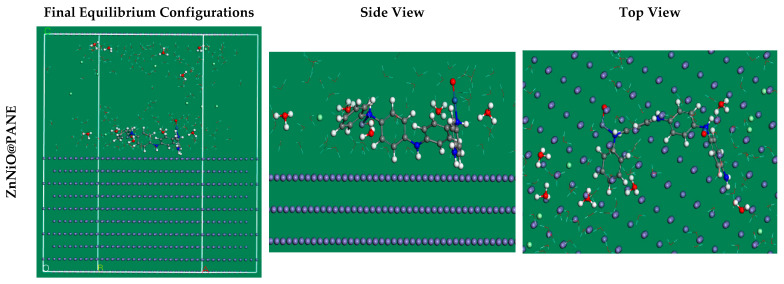
The highest suitable adsorption configuration based on the adsorption Locator module for the ZnNiO@PANE molecule on iron (110) Substrate.

In addition to providing a clear notion of the adsorption mechanism, MC simulations were used to identify the attractions of the inhibitor compounds with the metal interface via the adsorption locator module. Figure 12 demonstrates that the inhibitor molecules are existing in an approximately flat disposition and achieve the greatest appropriate adsorption measures for the prepared compounds on the CS alloy surface in acidic solution. This suggests an enhancement in the adsorption and extreme covered surface [72]. Additionally, Table 4 reveals the calculated results based on MC simulations for the adsorption energies. It was discovered that the ZnNiO@PANE molecule (−2002.0 kcal mol^−1^) has a higher negative value of adsorption energy than the PANE (−1831.61 kcal mol^−1^) and ZnO@PANE (−1938.63 kcal mol^−1^), which assumes energetic adsorption of ZnNiO@PANE on the metal substrate, constructing a stable adsorbed layer, and shielding the CS alloy interface from corrosion. These outcomes assent with the results of the experiment [73]. In addition, Table 4 exhibits that the value of adsorption energy for the ZnNiO@PANE compound for the pre-geometry optimization stage, i.e., unrelaxed (−1642.67 kcal mol^−1^) is more-negative than individual polymer and ZnO@PANE compounds (−1533.32 and −1589.97 kcal mol^−1^), as well as for post-geometry optimization stage, i.e*.,* relaxed (−359.38 kcal mol^−1^), which is also more negative adsorption energy than the corresponding values for PANE and ZnO@PANE compounds (−298.41 and −273.63 kcal mol^−1^), confirming a higher inhibition capability for ZnNiO@PANE composite compared with the other two compounds.

If adsorbed H_2_O or an inhibitor molecule has not been included, the d*E*_*ads*_/d*N*_i_ values help to clarify the steel/adsorbates conformation energy [73]. According to Table 4, the ZnNiO@PANE molecule exhibits superior adsorption over PANE and ZnO@PANE molecules because its d*E*_*ads*_/d*N*_i_ value (−298.32 kcal mol^−1^) surpasses those of PANE and ZO@PANE molecules (−219.81 and −273.21 kcal mol^−1^). Additionally, for water molecules, hydronium ions, and chloride ions, the d*E*_*ads*_/d*N*_i_ values are approximately −17.50, −54.43, and −96.03 kcal mol^−1^, respectively. These values are low when compared with the values of PANE molecules, ZnO@PANE and ZnNiO@PANE composites, revealing that inhibitor molecules adsorb strongly relative to H_2_O molecules, chloride ions, and hydronium ions, thereby promoting the inhibitor compound’s superiority over those ions. According to both experimental and theoretical investigations, the PANE, ZnO@PANE, and ZnNiO@PANE compounds are therefore stubbornly adsorbed on the CS alloy surface and create a potent adsorbed defensive film that leads to corrosion protection for CS alloy sample in the corrosive medium.

### 3.5. Comparative Studies with Previous Reports

This study is the first of its kind to use a nanocomposite based on ZnNiO NPs @PANE as a protective film for MS alloy in a corrosive media of HCl/NaCl. Table 5 compares investigations of the steel protection capabilities of ZnNiO@PANE nanocomposites with those of other polymeric nanocomposites utilized in an acidic chloride medium [74,75,76,77]. When compared to other materials, the ZnNiO@PANE nanocomposite has the best protective efficacy against acidic MS alloy corrosion.

## 4. Conclusions

In this work, novel protective films based on binary ZnO/NiO@polyaniline (ZnNiO@PANE) nanocomposite were successfully synthesized and confirmed. In line with the experiential and theoretical examinations, the following conclusions were made as a result:

1. The spectroscopic characterization methods, including DLS, UV-Vis, FTIR, and XPS approaches, in addition to other physicochemical methods, including XRD, HR-TEM, and FE-SEM confirmed the ZnO/NiO@polyaniline nanocomposite structure.
2. ZnO/NiO@PANE has shown to be an excellent corrosion inhibitor for MS alloy in HCl/NaCl solution. The protective capacity of the research improved in line with the increase in ZnO/NiO@PANE, reaching its maximum value of 97.89% at 200 ppm.
3. PDP experiments showed that the mixed-type ZnO/NiO@PANE nanocomposite inhibitor significantly blocked both the anodic and cathodic reactions.
4. The adsorption mechanism corresponds to the isotherm model of Langmuir. This seems to indicate that either the MS alloy surface is directly affected by the ZnNiO NPs or that the ZnNiO NPs promote the input of additional PANE active sites to the adsorption process.
5. ZnO/NiO@PANE demonstrated a particular attraction to adsorb onto the MS alloy substrate, which results in the construction of a shielding film, based on the DFT calculations.
6. MC simulations also revealed that the produced inhibitor molecules’ adsorption energies followed the sequence ZnO/NiO@PANE composite > ZnO@PANE > PANE polymer, which firmly supported the empirical results.

## Figures and Tables

**Table 1:** Potentiodynamic restrictions of MS-alloy corrosion in the blank HCl-containing brine solution and with the addition of different doses of ZnNiO@PANE at 298 K.

Inhibitors	*C*_*inh*_/ mg/L	*i*_*cor*_/ µA cm^−2^ ±SD	*−E*_*cor*_/ mV (Ag/AgCl)	*β*_a_/ mV dec ^−1^	*−β*_c_/ mV dec ^−1^	*θ*	*η*_t_/%
Blank	0.0	1254.34	439 ± 21	81.21	178.59	-	-
PANE	200	192.66	446 ± 23	87.41	182.08	0.846	84.64
ZnNiO@PANE	25	848.18	437 ± 22	81.91	175.35	0.323	32.38
	50	738.05	434 ± 15	79.53	182.38	0.411	41.16
	75	504.49	440 ± 17	84.37	177.59	0.597	59.78
	100	277.58	444 ± 19	88.67	179.29	0.778	77.87
	150	94.45	445 ± 16	86.46	178.38	0.924	92.47
	200	26.46	450 ± 13	87.83	182.71	0.978	97.89

**Table 2:** EIS parameters of M-alloy corrosion in the blank HCl-containing brine and with the addition of diverse concentrations of ZnNiO@PANE at 298 K.

Additive Codes	*C*_*inh*_/ mg/L	*R*_s_/ Ω cm^2^	*R_P_*/ Ω cm^2^ ±SD	*C_dl_*/ μF cm^−2^	*Q*_*CPE*_		*χ*^2^ × 10^−4^	*θ*	*η*_E_/%
					*Y*_0_/ μΩ^−1^ s^n^ cm^−2^	*n*			
Blank	0.0	0.59	56.1 ± 3.8	508.04	64.438	0.714	4.96	--	--
PANE	200	1.32	391.8 ± 23.2	90.879	9.911	0.804	4.16	0.840	84.05
ZnNiO@PANE	25	1.26	99.1 ± 8.3	162.14	30.921	0.857	4.88	0.433	43.39
	50	2.98	146.6 ± 12.1	133.96	22.187	0.815	4.52	0.617	61.73
	75	3.56	257.2 ± 21.8	83.88	16.302	0.841	5.33	0.781	78.18
	100	4.17	412.9 ± 32.5	45.16	11.913	0.834	5.45	0.864	86.41
	150	4.36	560.7 ± 29.8	26.70	9.603	0.813	4.73	0.899	89.99
	200	4.48	805.7 ± 42.6	15.049	7.194	0.831	4.15	0.930	93.03

**Table 3:** DFT parameters of the studied compounds.

Parameters	PANE	ZnO@PANE	ZnNiO@PANE
*E*_*HOMO*/_eV	−4.93	−4.51	−4.38
*E*_*LUMO*/_eV	−1.87	−2.03	−2.51
Δ*E* = *E*_*LUMO*_ − *E*_*HOMO*/_eV	3.06	2.46	1.87
µ/Debye	7.06	12.72	22.41
*η*	1.53	1.25	0.94
Δ*E*_*back-donation*_	−0.38	−0.31	−0.23
ω	3.76	4.28	6.35
Δ*N*	1.19	1.51	1.90
χ	3.41	3.28	3.45
σ	0.66	0.81	1.07

**Table 4:** Information and parameters derived from MC simulations of the PANE, ZnO@PANE, and ZnNiO@PANE compounds adherent to Iron (110).

Corrosion Systems	Adsorption Energy/ kcal mol^−1^	Rigid Adsorption Energy/ kcal mol^−1^	Deformation Energy/ kcal mol^−1^	d*E*_*ads*_/dN_i_: Inhibitor kcal mol^−1^	d*E*_*ads*_/d*N*_i_: Cl^−^ Ions kcal mol^−1^	d*E*_*ads*_/d*N*_i_: Hydronium kcal mol^−1^	d*E*_*ads*_/d*N*_i_: Water kcal mol^−1^
Fe (110)	−1831.61	−1533.32	−298.41	−219.81	−95.62	−54.05	−17.26
PANE							
Water							
Hydronium							
Cl^−^ ions							
Fe (110)	−1938.63	−1589.97	−348.63	−273.21	−96.12	−54.90	−17.76
ZnO@PANE							
Water							
Hydronium							
Cl^−^ ions							
Fe (110)	−2002.05	−1642.67	−359.38	−298.32	−96.35	−54.34	−17.47
ZnNiO@PANE							
Water							
Hydronium							
Cl^−^ ions							

**Table 5:** Comparative studies with previous reports.

System	Metal/Alloys	Techniques Used	Protection Capacity	References
ZnNiO@PANE nanocomposite	MS alloy	SEM, TEM, XPS, FTIR, XRD, E_ocp_, PDP, and EIS	97.89	Current work
Hybrid ZnO and NiO Nanoparticles	Carbon steel	EIS, PDP, SEM	96.28	[74]
Nickel oxide (NiO) epoxy nanocomposite	Mild steel	DLS, FTIR, AFM, SEM	85.34	[75]
Zn–NiO composite	Mild steel	TGA, XPS, EIS, SEM/EDX, TEM, FTIR,	87.02	[76]
NiO–epoxy nanocomposite coatings	Mild steel	SECM, FTIR, TGA, EIS, FE-SEM, FIB-TEM_,_ TEM/EDX	93.23	[77]

## Data Availability

The raw/processed data generated in this work are available upon request from the corresponding author.

## Supplementary Materials

The following supporting information can be downloaded at: https://www.mdpi.com/article/10.3390/polym14214734/s1, Figure S1: A representative scheme of the fabrication route of ZnO nanoparticles; Figure S2: A representative scheme of the fabrication route of ZnO-NiO nanoparticles; Figure S3: A representative scheme of the fabrication route of binary MO/PANE nanocomposites by oxidation in situ-polymerization; Table S1: The evaluated Fukui indices.
