# Supplementary material for: Advanced Protective Films Based on Binary ZnO-NiO@polyaniline Nanocomposite for Acidic Chloride Steel Corrosion: An Integrated Study of Theoretical and Practical Investigations
Source: Polymers (Basel). 2022 Nov 4;14(21):4734. doi: 10.3390/polym14214734 (PMC9658172; doi:10.3390/polym14214734)
Supplement: Supplementary file 1 [file polymers-14-04734-s001.zip › polymers-1976519-supplementary.pdf]

polymers
MDPI

Supplementary Materials

Advanced Protective Films Based on Binary ZnO-NiO@polyaniline Nanocomposite for Acidic Chloride Steel Corrosion: An Integrated Study of Theoretical and Practical Investigations

May Ahmed Al-Masoud [1], Mai M. Khalaf [1,2], Fakiha El-Taib Heakal [3,*], Mohamed Gouda [1,*], Ibrahim M. A. Mohamed [2], Kamal Shalabi [4,5] and Hany M. Abd El-Lateef [1,2,*]

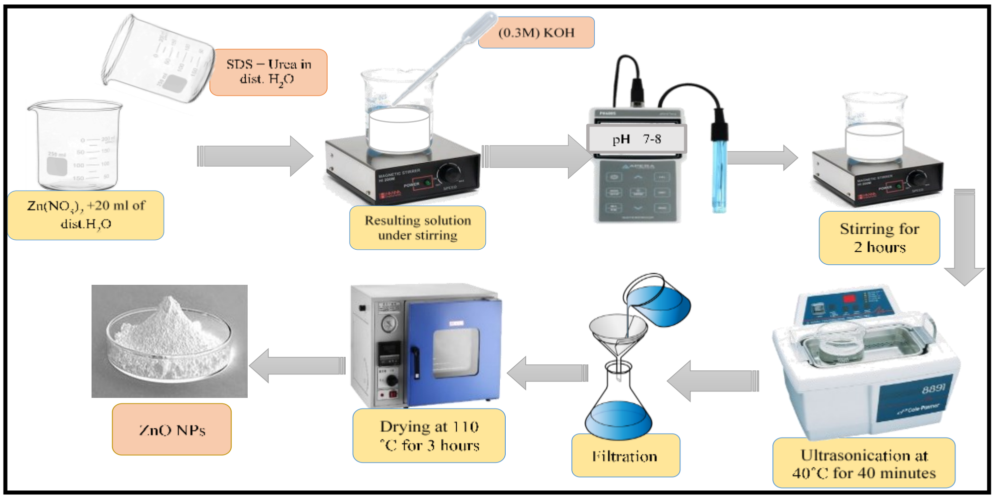

Figure S1. A representative scheme of the fabrication route of ZnO nanoparticles.

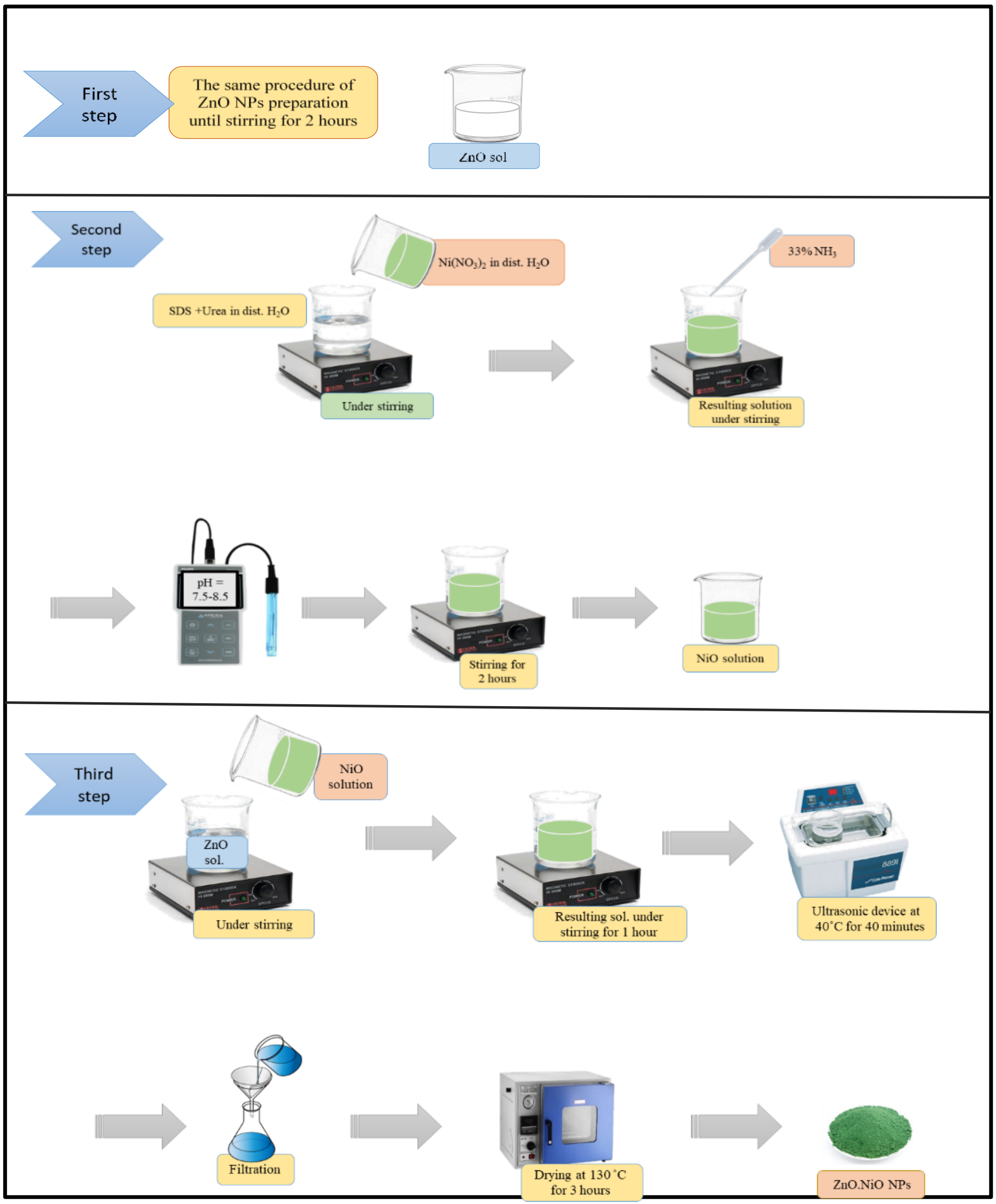

Figure S2. A representative scheme of the fabrication route of ZnO-NiO nanoparticles.

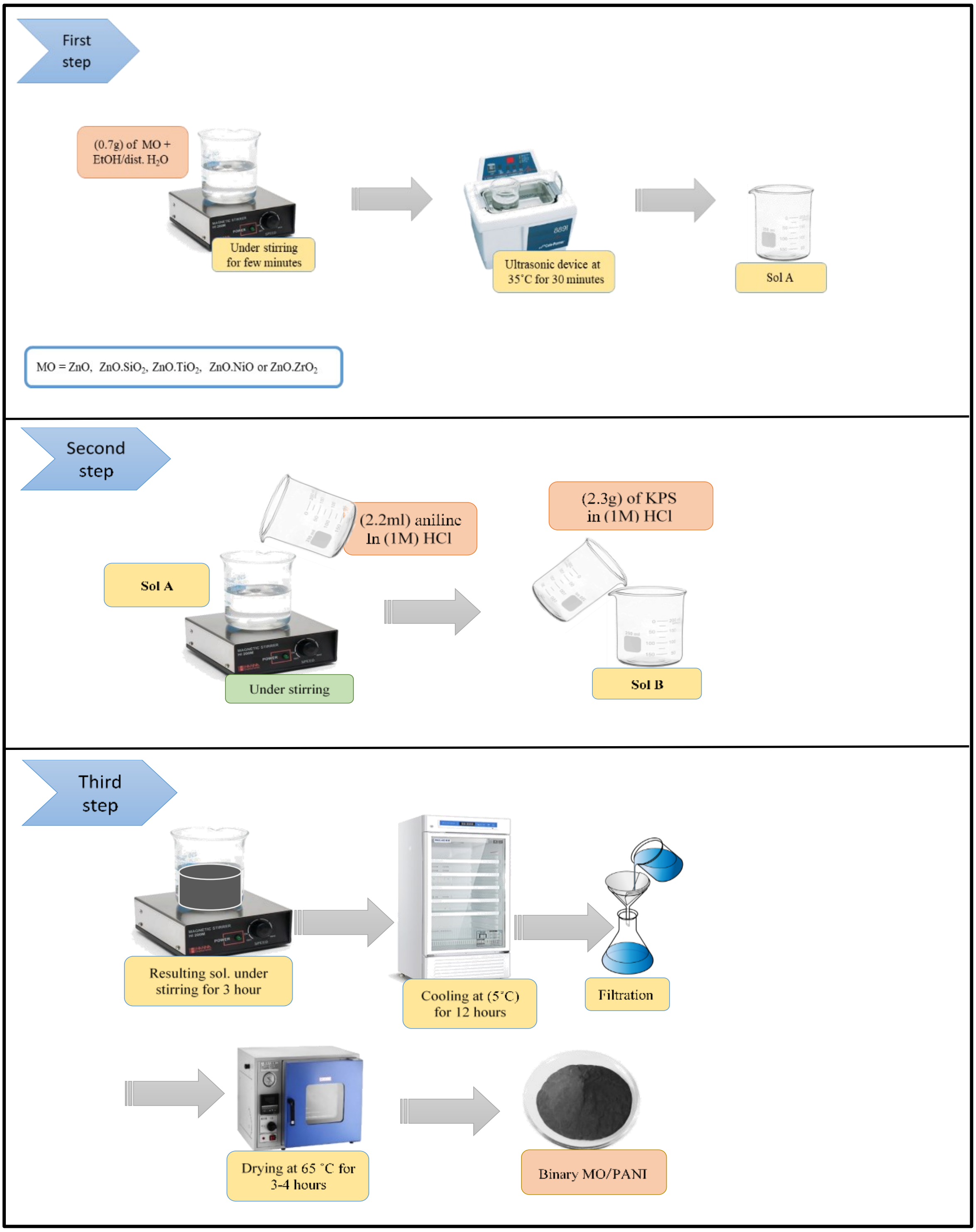

Figure S3. A representative scheme of the fabrication route of binary MO/PANE nanocomposites by oxidation in situ-polymerization.

Table S1. The evaluated Fukui indices.

Atom No	PANE						
	f+	ω+	σ+	f-	ω-	σ-	Mulliken Charges
C1	0.027	0.101	0.018	0.005	0.019	0.003	-0.087
C2	0.026	0.098	0.017	0.005	0.019	0.003	-0.043
C3	0.030	0.113	0.020	0.006	0.023	0.004	-0.045
C4	0.024	0.090	0.016	0.005	0.019	0.003	-0.042
C5	0.030	0.113	0.020	0.006	0.023	0.004	-0.091
C6	0.019	0.071	0.012	0.001	0.004	0.001	0.203
N7	-0.004	-0.015	-0.003	-0.001	-0.004	-0.001	-0.244
C8	0.023	0.086	0.015	0.025	0.094	0.016	0.169
C9	0.032	0.120	0.021	0.014	0.053	0.009	-0.083
C10	0.034	0.128	0.022	0.026	0.098	0.017	-0.101
C11	0.027	0.101	0.018	0.011	0.041	0.007	0.222
C12	0.028	0.105	0.018	0.022	0.083	0.014	-0.130
C13	0.033	0.124	0.022	0.015	0.056	0.010	-0.086
N14	0.008	0.030	0.005	0.046	0.173	0.030	-0.270
C15	0.023	0.086	0.015	0.014	0.053	0.009	0.223
C16	0.027	0.101	0.018	0.026	0.098	0.017	-0.102
C17	0.030	0.113	0.020	0.016	0.060	0.010	-0.082
C18	0.022	0.083	0.014	0.019	0.071	0.012	0.169
C19	0.025	0.094	0.016	0.016	0.060	0.010	-0.079
C20	0.028	0.105	0.018	0.026	0.098	0.017	-0.135
N21	-0.002	-0.008	-0.001	-0.002	-0.008	-0.001	-0.242
C22	0.005	0.019	0.003	0.041	0.154	0.027	0.148
C23	0.012	0.045	0.008	0.019	0.071	0.012	-0.079
C24	0.018	0.068	0.012	0.048	0.180	0.031	-0.124
C25	0.007	0.026	0.005	0.018	0.068	0.012	0.231
C26	0.017	0.064	0.011	0.044	0.165	0.029	-0.122
C27	0.013	0.049	0.008	0.021	0.079	0.014	-0.081
N28	0.02	0.075	0.013	0.098	0.368	0.064	-0.352
Atom no	ZnO@PANE						
	f+	ω+	σ+	f-	ω-	σ-	Mulliken Charges
C1	0.024	0.103	0.019	0.005	0.021	0.004	-0.096
C2	0.023	0.098	0.018	0.005	0.021	0.004	-0.067
C3	0.027	0.116	0.022	0.006	0.026	0.005	-0.069
C4	0.020	0.086	0.016	0.004	0.017	0.003	-0.066
C5	0.028	0.120	0.022	0.006	0.026	0.005	-0.092
C6	0.017	0.073	0.014	0.002	0.009	0.002	0.162
N7	-0.011	-0.047	-0.009	-0.009	-0.039	-0.007	-0.301
C8	0.017	0.073	0.014	0.015	0.064	0.012	0.114
C9	0.031	0.133	0.025	0.013	0.056	0.010	-0.082
C10	0.028	0.120	0.022	0.020	0.086	0.016	-0.114
C11	0.025	0.107	0.020	0.011	0.047	0.009	0.184

C12	0.030	0.128	0.024	0.022	0.094	0.018	-0.159
C13	0.027	0.116	0.022	0.011	0.047	0.009	-0.086
N14	0.007	0.030	0.006	0.031	0.133	0.025	-0.237
C15	0.024	0.103	0.019	0.011	0.047	0.009	0.177
C16	0.029	0.124	0.023	0.019	0.081	0.015	-0.112
C17	0.030	0.128	0.024	0.013	0.056	0.010	-0.080
C18	0.023	0.098	0.018	0.017	0.073	0.014	0.128
C19	0.027	0.116	0.022	0.011	0.047	0.009	-0.094
C20	0.028	0.120	0.022	0.020	0.086	0.016	-0.155
N21	-0.005	-0.021	-0.004	-0.004	-0.017	-0.003	-0.298
C22	0.003	0.013	0.002	0.017	0.073	0.014	0.114
C23	0.014	0.060	0.011	0.010	0.043	0.008	-0.093
C24	0.014	0.060	0.011	0.021	0.090	0.017	-0.120
C25	0.008	0.034	0.006	0.011	0.047	0.009	0.148
C26	0.015	0.064	0.012	0.021	0.090	0.017	-0.121
C27	0.013	0.056	0.010	0.011	0.047	0.009	-0.084
N28	0.013	0.056	0.010	0.044	0.188	0.035	-0.368
Zn29	0.044	0.188	0.035	0.199	0.851	0.160	-0.015
Zn30	0.037	0.158	0.030	0.136	0.582	0.109	0.015
O31	0.001	0.004	0.001	0.010	0.043	0.008	-0.550
O32	0.001	0.004	0.001	0.006	0.026	0.005	-0.555
Atom no	**ZnNiO@PANE**						
	f+	ω+	σ+	f-	ω-	σ-	Mulliken Charges
C1	0.072	0.457	0.077	0.064	0.406	0.068	-0.091
C2	0.072	0.457	0.077	0.064	0.406	0.068	-0.062
C3	0.100	0.635	0.107	0.088	0.559	0.094	-0.074
C4	0.072	0.457	0.077	0.064	0.406	0.068	-0.062
C5	0.088	0.559	0.094	0.072	0.457	0.077	-0.090
C6	0.032	0.203	0.034	0.024	0.152	0.026	0.177
N7	-0.040	-0.254	-0.043	-0.032	-0.203	-0.034	-0.579
C8	0.132	0.838	0.141	0.120	0.762	0.128	0.191
C9	0.048	0.305	0.051	0.040	0.254	0.043	-0.085
C10	0.155	0.986	0.166	0.144	0.914	0.154	-0.090
C11	0.088	0.559	0.094	0.080	0.508	0.086	0.177
C12	0.133	0.843	0.142	0.120	0.762	0.128	-0.122
C13	0.088	0.559	0.094	0.080	0.508	0.086	-0.068
N14	0.224	1.422	0.240	0.240	1.524	0.257	-0.171
C15	0.112	0.711	0.120	0.104	0.660	0.111	0.191
C16	0.154	0.978	0.165	0.152	0.965	0.163	-0.074
C17	0.088	0.559	0.094	0.080	0.508	0.086	-0.042

C18	0.048	0.305	0.051	0.040	0.254	0.043	0.085
C19	0.080	0.508	0.086	0.072	0.457	0.077	-0.056
C20	0.146	0.927	0.156	0.136	0.864	0.146	-0.120
N21	-0.016	-0.102	-0.017	-0.008	-0.051	-0.009	-0.408
C22	0.216	1.372	0.231	0.208	1.321	0.223	0.204
C23	0.112	0.711	0.120	0.120	0.762	0.128	-0.067
C24	0.216	1.372	0.231	0.208	1.321	0.223	-0.093
C25	0.132	0.838	0.141	0.120	0.762	0.128	0.174
C26	0.224	1.422	0.240	0.216	1.372	0.231	-0.095
C27	0.056	0.356	0.060	0.064	0.406	0.068	-0.102
N28	0.368	2.337	0.394	0.400	2.540	0.428	-0.239
Zn29	0.640	4.064	0.685	0.680	4.318	0.728	0.873
Ni30	0.760	4.826	0.813	0.792	5.029	0.847	0.585
O31	0.656	4.166	0.702	0.680	4.318	0.728	-0.770
O32	0.656	4.166	0.702	0.688	4.369	0.736	-0.644
